# Comparative value of metabolic and inflammatory indices for identifying OCT-derived coronary plaque vulnerability in patients with acute coronary syndrome and type 2 diabetes mellitus

**DOI:** 10.3389/fcvm.2026.1892356

**Published:** 2026-07-29

**Authors:** Yulong Liu, Xin Yang, Yang Zhang, Yaling Wang

**Affiliations:** 1 Department of Cardiology, The Second Hospital of Hebei Medical University, Shijiazhuang, Hebei, China; 2 Department of Cardiac Surgery, Shijiazhuang People’s Hospital, Shijiazhuang, Hebei, China

**Keywords:** acute coronary syndrome, optical coherence tomography, plaque vulnerability, thin-cap fibroatheroma, triglyceride-glucose index, type 2 diabetes mellitus

## Abstract

**Background/objectives:**

Patients with acute coronary syndrome (ACS) and type 2 diabetes mellitus (T2DM) are prone to coronary plaque vulnerability and recurrent ischemic events. Although optical coherence tomography (OCT) enables a detailed assessment of vulnerable plaque features, it is invasive and not routinely applicable in all patients. Several routinely available metabolic and inflammatory indices have been associated with cardiovascular risk; however, their comparative value in identifying OCT-defined vulnerable plaques in patients with ACS and T2DM remains unclear.

**Methods:**

This single-center retrospective study included 115 patients with ACS and T2DM who underwent OCT assessment of one target coronary lesion between July 2024 and May 2025 at our institution. Candidate biomarkers included the triglyceride-glucose (TyG) index, fasting blood glucose (FBG), triglycerides (TG), atherogenic index of plasma (AIP), TG/high-density lipoprotein cholesterol (TG/HDL-C) ratio, glycated hemoglobin (HbA1c), and selected inflammatory indices. The primary outcome was OCT-defined thin-cap fibroatheroma (TCFA), and fibrous cap thickness (FCT) was analyzed as a key continuous OCT parameter. Logistic regression, linear regression, and receiver operating characteristic analyses were performed to compare the associations and discriminative abilities of the candidate biomarkers.

**Results:**

TCFA was identified in 63 (54.8%) of the 115 patients. Compared with patients without TCFA, those with TCFA had higher levels of TG, FBG, HbA1c, TyG, AIP, and TG/HDL-C, whereas the neutrophil-to-lymphocyte ratio and systemic immune-inflammation index did not differ significantly between the groups. After multivariable adjustment, TyG index showed the most consistent association with TCFA among the evaluated biomarkers (odds ratio per 1-SD increase, 4.42; 95% confidence interval, 2.34−8.35; *P* < 0.001). TyG was also inversely associated with FCT, a continuous component underlying TCFA classification (adjusted beta per 1-SD increase, −19.0 μm; 95% confidence interval, −25.5 to −12.4 μm; *P* < 0.001). In exploratory single-biomarker receiver operating characteristic analysis, TyG had the largest numerical discriminative estimate for TCFA (area under the curve, 0.792; 95% confidence interval, 0.711−0.874), followed by FBG, TG, AIP, and TG/HDL-C. Inflammatory indices showed limited ability to discriminate TCFA.

**Conclusions:**

Among the routinely available metabolic and inflammatory indices, TyG index showed the most consistent association with OCT-defined coronary plaque vulnerability in patients with ACS and T2DM and had the largest numerical AUC in exploratory single-biomarker discrimination analyses. These findings suggest that TyG is an accessible metabolic correlate of OCT-defined plaque vulnerability in this high-risk cohort; however, larger prospective studies are needed for confirmation.

## Introduction

Acute coronary syndrome remains one of the most clinically important manifestations of atherosclerotic cardiovascular disease (1, 2). Contemporary guidelines emphasize rapid diagnosis, invasive assessment when indicated, and secondary prevention; however, the risk of recurrent ischemia remains substantial in metabolically high-risk patients (2). Coronary plaque vulnerability, particularly thin-cap fibroatheroma, plaque rupture, lipid-rich morphology, and inflammatory cell infiltration, plays a central role in the pathophysiology of acute coronary events (3, 4).

Type 2 diabetes mellitus is closely linked to accelerated atherosclerosis, lipid-rich plaque formation, endothelial dysfunction and heightened inflammatory activity (5–7). Therefore, patients with ACS and T2DM represent a particularly high-risk phenotype in whom the characterization of plaque vulnerability may have clinical and mechanistic relevance (6, 7). OCT provides high-resolution intracoronary imaging and allows detailed assessment of FCT, TCFA, plaque rupture, macrophage infiltration, microvessels/microchannels, calcification, cholesterol crystals and thrombus (3, 4). However, OCT is invasive, resource-intensive, and not routinely applicable to all patients.

Metabolic and inflammatory biomarkers derived from routine laboratory tests may provide accessible information related to plaque vulnerability in patients with CAD. The TyG index, calculated from fasting triglyceride and glucose levels, has been widely used as a surrogate marker of insulin resistance (8, 9). AIP and TG/HDL-C reflect atherogenic lipid patterns (10, 11). Inflammatory indices were included as exploratory comparators, with prior work supporting systemic immune-inflammatory indices and OCT vulnerability associations (12–14).

Previous OCT studies have evaluated TyG, AIP, and inflammatory indices in ACS or coronary artery disease populations (13–16). OCT studies have also linked dysglycemia or diabetes-related characteristics with vulnerable plaque features in ACS or AMI populations (6, 7). Nevertheless, most studies have focused on a single biomarker, and comparative evidence in patients with ACS and T2DM remains limited. Because T2DM is characterized by combined glucose and lipid metabolic disturbances, a head-to-head comparison of routinely available metabolic and inflammatory indices may help clarify which marker is most closely associated with OCT-defined plaque vulnerability.

This study aimed to compare the value of metabolic and inflammatory indices in identifying OCT-derived coronary plaque vulnerability in patients with ACS and T2DM. The primary outcome was OCT-defined TCFA, and FCT was analyzed as a key continuous outcome.

## Materials and methods

### Study design and population

This was a single-center, retrospective observational study. During the study period, 181 consecutive patients with ACS and T2DM who underwent OCT assessment before definitive PCI treatment between July 2024 and May 2025 were screened. Patients who underwent balloon pre-dilation before OCT imaging were excluded (*n* = 29). The remaining 152 patients constituted the qualifying pre-intervention OCT cohort. After further exclusion of patients with poor-quality OCT images, missing key clinical or laboratory data, non-evaluable OCT indices, documented familial hypertriglyceridemia, or other eligibility reasons, 115 patients were included in the final analytic cohort.

**Figure 1:**
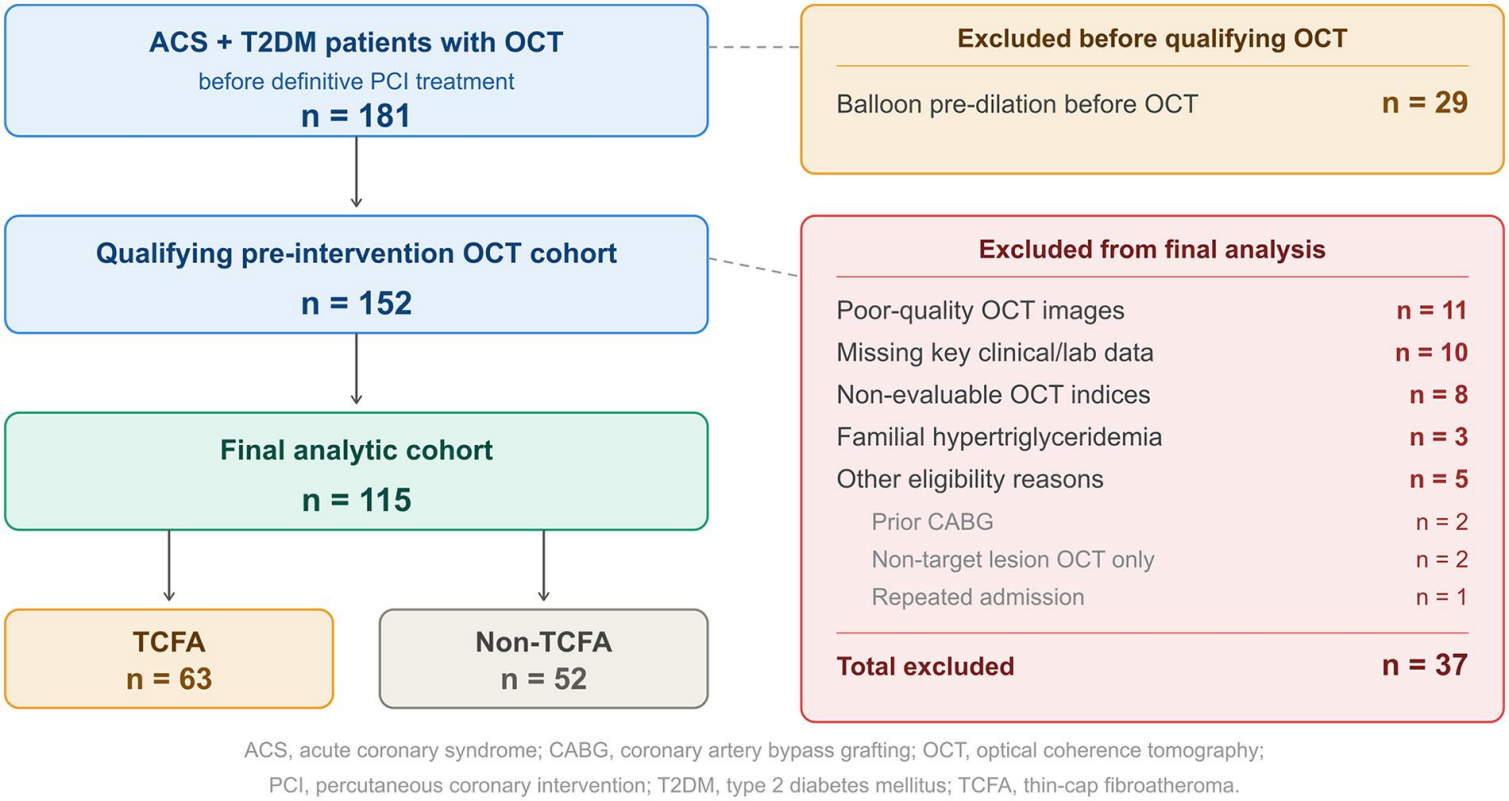
Study flowchart. During the study period, 181 patients with ACS and T2DM who underwent OCT assessment before definitive PCI treatment were screened. Patients who underwent balloon pre-dilation before OCT imaging were excluded (*n* = 29), leaving 152 patients in the qualifying pre-intervention OCT cohort. After further exclusions, 115 patients were included in the final analysis and were classified according to OCT-defined TCFA status. ACS, acute coronary syndrome; OCT, optical coherence tomography; PCI, percutaneous coronary intervention; TCFA, thin-cap fibroatheroma; T2DM, type 2 diabetes mellitus.

The study flowchart is shown in Figure 1.

The inclusion criteria were as follows: (1) diagnosis of ACS; (2) diagnosis of T2DM; (3) coronary angiography and PCI with OCT imaging of the target lesion before balloon pre-dilation; and (4) complete clinical, laboratory, and OCT data required for the main analyses. The exclusion criteria were as follows: (1) poor-quality OCT images precluding reliable plaque assessment; (2) missing key clinical or laboratory data; (3) non-evaluable OCT indices; (4) documented familial hypertriglyceridemia; and (5) other eligibility reasons, including prior coronary artery bypass grafting, OCT imaging of non-target lesions only, and repeated admission during the study period. Balloon pre-dilation before OCT imaging was an additional exclusion applied before the qualifying OCT cohort and is reported separately in the patient flow (Figure 1).

Each patient contributed one target lesion. The target lesion was defined as the clinically identified culprit lesion for the index ACS event, which underwent PCI and was assessed by OCT before balloon pre-dilation. Culprit lesion identification was made during routine clinical care by the treating interventional team based on an integrated assessment of clinical presentation, electrocardiographic findings, coronary angiographic lesion morphology, relevant imaging information when clinically applicable, and intraprocedural judgment. This study was approved by the Ethics Committee of the Second Hospital of Hebei Medical University (approval number: 2021-R375). The requirement for written informed consent was waived by the ethics committee because of the retrospective design and use of de-identified data.

Because the final analytic cohort was restricted to patients with complete clinical, laboratory, and OCT data required for the main analyses, a complete-case analysis was performed, and no imputation was applied.

### Clinical and laboratory data

Demographic characteristics, medical history, cardiovascular risk factors, laboratory data, angiographic findings, and procedural information were extracted from the electronic medical record system and procedural documentation. The clinical variables included age, sex, body mass index (BMI), current smoking, hypertension, hyperlipidemia, previous myocardial infarction, ACS presentation, culprit target-vessel location, arterial access route, and PCI strategy. Laboratory variables included total cholesterol (TC), TG, high-density lipoprotein cholesterol (HDL-C), low-density lipoprotein cholesterol (LDL-C), FBG, HbA1c, white blood cell differential counts, platelet count, albumin, and high-sensitivity C-reactive protein (hs-CRP).

Laboratory data were obtained during the index hospitalization. Lipid variables and FBG used for TyG and lipid-related indices were obtained from fasting blood samples, preferentially from the first available fasting morning sample during the index hospitalization. For patients undergoing emergency PCI before fasting laboratory sampling, the first available fasting morning sample after clinical stabilization was used. LDL-C was directly measured by the hospital laboratory. Complete blood counts used for inflammatory indices were obtained from baseline or early hospitalization blood tests recorded in the electronic medical record.

Current smoking status was defined according to the documentation at admission. Hypertension, hyperlipidemia, previous myocardial infarction, ACS, and T2DM were identified based on medical records and standard clinical diagnostic criteria (2, 5). ACS presentation was classified as ST-segment elevation myocardial infarction (STEMI), non-ST-segment elevation myocardial infarction (NSTEMI), or unstable angina according to the clinical diagnosis documented during the index hospitalization. Culprit target-vessel location referred to the vessel containing the clinically identified culprit lesion assessed by OCT.

### Biomarker definitions

The TyG index was calculated using fasting TG and fasting blood glucose as follows (8, 9):

TyG = ln [TG (mg/dL) × FBG (mg/dL)/2]

When TG and FBG were recorded in mmol/L, they were converted to mg/dL before the calculations. The AIP was calculated as follows (10):

AIP = log10 [TG/HDL-C]

using triglycerides (TG) and high-density lipoprotein cholesterol (HDL-C) levels in mmol/L. The TG/HDL-C ratio was calculated using the same lipid unit system (11). Exploratory inflammatory indices were calculated from neutrophil, lymphocyte, platelet, monocyte, and HDL-C values using predefined arithmetic ratios (12–14):

NLR = neutrophil count/lymphocyte count

PLR = platelet count/lymphocyte count

SII = platelet count   ×   neutrophil count/lymphocyte count

SIRI = neutrophil count   ×   monocyte count/lymphocyte count

PIV = neutrophil count   ×   platelet count   ×   monocyte count/lymphocyte count

MHR = monocyte count/HDL-C

For the primary comparative analysis, the core biomarker set included TyG, FBG, TG, AIP, TG/HDL-C and HbA1c. Inflammatory indices were analyzed as exploratory variables.

### OCT image acquisition and analysis

OCT imaging was performed using the OPTIS Next system (Abbott, USA) before balloon pre-dilation in the qualifying cohort. Patients who underwent balloon pre-dilation before OCT imaging were excluded. In patients with impaired coronary flow or occlusive thrombus that prevented safe OCT catheter passage or adequate image acquisition, manual thrombus aspiration or flow restoration before OCT was permitted when clinically necessary. All eligible OCT images were analyzed offline using the dedicated software. OCT image acquisition, quantitative measurements, and reporting were performed according to established OCT standards, including the updated 2025 OCT/OFDI expert consensus where applicable (3, 4, 17). Qualitative plaque features were identified according to established OCT criteria (3, 4).

Two independent experienced OCT readers, blinded to laboratory data and biomarker values, independently assessed the predefined target OCT images. Agreement for TCFA and lipid arc classification was evaluated using Cohen's kappa. Reproducibility for continuous FCT measurements was evaluated using a two-way random-effects, single-measure, absolute-agreement intraclass correlation coefficient. Disagreements between the two readers were resolved by a third experienced OCT reader, and the final consensus classification was used for all analyses.

The minimum lumen area (MLA) and minimum lumen diameter (MLD) were measured at the most stenotic cross-section after longitudinal reconstruction. FCT was measured at the thinnest part of the fibrous cap overlying a lipid plaque and is reported in micrometers (*μ*m). TCFA was defined as a lipid-rich plaque with a minimum FCT <65 μm; in this study, lipid-rich plaques were operationally identified by a lipid arc ≥180°. OCT-defined macrophage signal was identified as punctate or band-like high-intensity regions with signal attenuation. Microvessels/microchannels were defined as no-signal tubuloluminal or cavity-like structures without connection to the vessel lumen, visible in multiple contiguous cross-sections. Calcification was defined as a sharply bordered, low-signal region. Layered plaques were defined as one or more layers with different optical densities and clear demarcation from the underlying plaque components. Cholesterol crystals were identified as thin, linear, and high-intensity regions within the plaque. Thrombus and plaque ruptures were identified according to the standard OCT criteria (3, 4).

### Outcomes

The primary outcome was the OCT-defined TCFA. The key continuous OCT outcome was the FCT. Secondary OCT features included MLA, MLD, area stenosis, plaque rupture, erosion, macrophage infiltration, microvessels/microchannels, calcification, layered plaque, cholesterol crystals and thrombus.

### Statistical analysis

Continuous variables are presented as medians (interquartile range), and categorical variables are presented as numbers (%). Between-group comparisons according to TCFA status were performed using Wilcoxon rank-sum tests for continuous variables and Fisher's exact or chi-square tests for categorical variables, as appropriate.

Logistic regression was used to assess the association between candidate biomarkers and TCFA. Continuous biomarkers were standardized, and odds ratios were reported per 1-SD increase. Linear regression and Spearman's correlation analyses were used to assess the associations between candidate biomarkers and FCT. Three model levels were used: Model 1, unadjusted; Model 2, adjusted for age, sex, and BMI; and Model 3, additionally adjusted for current smoking status, LDL-C, and hypertension. Each standardized biomarker was entered into a separate regression model with the same covariate set to avoid collinearity among the glucose- and lipid-derived indices.

Receiver operating characteristic (ROC) curve analysis was used as an exploratory single-biomarker discrimination analysis of candidate biomarkers for OCT-defined TCFA. These analyses were not intended to develop or validate a clinical prediction model, and no optimal cut-off thresholds or calibration analyses were performed. Formal pairwise AUC comparisons were not performed; therefore, differences in AUCs were interpreted descriptively. To assess potential optimism in the apparent AUC estimates, bootstrap optimism correction with 1,000 resamples was performed as a sensitivity analysis for the prespecified core biomarkers (TyG, FBG, TG, AIP, TG/HDL-C, and HbA1c). Optimism-corrected AUCs are reported in Supplementary Table S1.

To address potential residual confounding, we performed additional sensitivity analyses for the association between TyG index and OCT-defined TCFA. Given the modest sample size, the primary multivariable model was kept parsimonious and adjusted for age, sex, BMI, current smoking, LDL-C, and hypertension. Sensitivity models added ACS presentation (STEMI, NSTEMI, or unstable angina), calculated eGFR, or log-transformed hs-CRP one at a time to the primary multivariable model. eGFR was calculated from age, sex, and serum creatinine using the CKD-EPI 2021 creatinine equation. These analyses were designed to examine the robustness of the main association, not to construct an expanded prediction model. The results of these sensitivity analyses are provided in Supplementary Table S2.

A two-sided *P* value <0.05 was considered statistically significant. Statistical analyses were performed using SPSS version 25.0 (IBM Corp., Armonk, NY, USA).

## Results

### Study population

A total of 115 patients with ACS and T2DM who underwent OCT assessment of one target coronary lesion were included in the final analysis. OCT-defined TCFA was identified in 63 patients (54.8%), whereas 52 patients (45.2%) did not have TCFA. ACS presentation included unstable angina in 75 patients (65.2%), STEMI in 29 patients (25.2%), and NSTEMI in 11 patients (9.6%).

### Baseline characteristics according to TCFA status

The baseline characteristics according to TCFA status are shown in Table 1. Age, sex distribution, BMI, hypertension, hyperlipidemia, previous myocardial infarction, ACS presentation, and culprit target-vessel location were reported in Table 1. Current smoking was more frequent in patients with TCFA than in those without TCFA (46.0% vs. 21.2%, *P* = 0.006).

**Table 1:** Baseline characteristics according to TCFA status.

Characteristic	Non-TCFA (*n* = 52)	TCFA (*n* = 63)	*P* value
Age, years	63 (53-70)	60 (54-68)	0.776
Male sex	31 (59.6%)	44 (69.8%)	0.326
Body mass index, kg/m^2^	25 (24-27)	26 (24-28)	0.489
ACS presentation			0.009
STEMI	6 (11.5%)	23 (36.5%)	
NSTEMI	6 (11.5%)	5 (7.9%)	
Unstable angina	40 (76.9%)	35 (55.6%)	
Culprit target-vessel location			0.435
LM	1 (1.9%)	0 (0.0%)	
LAD	31 (59.6%)	45 (71.4%)	
LCX	8 (15.4%)	8 (12.7%)	
RCA	12 (23.1%)	10 (15.9%)	
Current smoking	11 (21.2%)	29 (46.0%)	0.006
Hypertension	37 (71.2%)	50 (79.4%)	0.384
Hyperlipidemia	37 (71.2%)	48 (76.2%)	0.670
Previous myocardial infarction	14 (26.9%)	9 (14.3%)	0.106
eGFR, mL/min/1.73 m^2^	87 (74–95)	92 (78–101)	0.327
hs-CRP, mg/L	2.30 (1.50–4.15)	3.40 (2.10–5.05)	0.062
Total cholesterol, mmol/L	3.88 (3.20–4.86)	4.13 (3.60–4.98)	0.226
Triglycerides, mmol/L	1.50 (1.06–1.95)	2.26 (1.60–2.83)	<0.001
HDL-C, mmol/L	1.04 (0.91–1.14)	1.07 (0.97–1.23)	0.214
LDL-C, mmol/L	2.55 (2.10–3.23)	2.70 (2.27–3.37)	0.261
Fasting blood glucose, mmol/L	7.80 (6.70–8.90)	9.8 (8.2–11.2)	<0.001
HbA1c, %	6.95 (6.57–7.45)	7.30 (6.90–7.90)	0.023
TyG index	9.08 (8.77–9.44)	9.77 (9.36–9.99)	<0.001
AIP	0.14 (0.05–0.28)	0.30 (0.18–0.40)	<0.001
TG/HDL-C ratio	1.37 (1.12–1.88)	1.99 (1.50–2.54)	<0.001
NLR	2.42 (1.63–3.67)	2.64 (1.85–3.77)	0.524
SII	601 (400–849)	603 (381–1044)	0.980

Continuous variables are presented as median (interquartile range), and categorical variables as *n* (%). *P* values were calculated using Wilcoxon rank-sum tests or Fisher's exact or chi-square tests, as appropriate.

eGFR, estimated glomerular filtration rate; hs-CRP, high-sensitivity C-reactive protein; TyG, triglyceride-glucose index; AIP, atherogenic index of plasma; NLR, neutrophil-to-lymphocyte ratio; SII, systemic immune-inflammation index.

Patients with TCFA had higher levels of TG [2.26 (1.60−2.83) vs. 1.50 (1.06−1.95) mmol/L, *P* < 0.001], FBG [9.80 (8.25−11.20) vs. 7.80 (6.70−8.90) mmol/L, *P* < 0.001], HbA1c [7.30 (6.90−7.90) vs. 6.95 (6.57−7.45)%, *P* = 0.023], TyG [9.77 (9.36−9.99) vs. 9.08 (8.77−9.44), *P* < 0.001], AIP [0.30 (0.18−0.40) vs. 0.14 (0.05−0.28), *P* < 0.001], and TG/HDL-C ratio [1.99 (1.50−2.54) vs. 1.37 (1.12−1.88), *P* < 0.001]. In contrast, the NLR and SII did not differ significantly between the two groups. hs-CRP tended to be higher in patients with TCFA [3.4 (2.1−5.05) vs. 2.3 (1.5−4.15) mg/L], but the between-group difference did not reach statistical significance (*P* = 0.062).

Most procedures were performed through transradial access (108/115, 93.9%), with transfemoral access used in 7 patients (6.1%). Stent-based PCI was performed in 82 patients (71.3%), and DCB treatment was used in 33 patients (28.7%). Among 40 myocardial infarction patients, 35 underwent stent implantation during the index procedure, and 5 underwent staged stent implantation after TIMI 3 flow restoration. Pre-OCT thrombus aspiration was performed in 3 myocardial infarction patients.

Continuous variables are presented as median (interquartile range), and categorical variables as *n* (%). *P* values were calculated using Wilcoxon rank-sum tests, Fisher's exact tests, or chi-square tests, as appropriate. ACS presentation was classified according to the clinical diagnosis during the index hospitalization. Culprit target-vessel location refers to the vessel containing the clinically identified culprit lesion assessed by OCT. AIP, atherogenic index of plasma; FBG, fasting blood glucose; HDL-C, high-density lipoprotein cholesterol; hs-CRP, high-sensitivity C-reactive protein; LAD, left anterior descending artery; LCX, left circumflex artery; LDL-C, low-density lipoprotein cholesterol; LM, left main coronary artery; NLR, neutrophil-to-lymphocyte ratio; RCA, right coronary artery; SII, systemic immune-inflammation index; TCFA, thin-cap fibroatheroma; TG, triglycerides; TyG, triglyceride-glucose index.

### OCT characteristics according to TCFA status

The OCT characteristics according to TCFA status are presented in Table 2. Interobserver agreement was good for TCFA (Cohen's kappa = 0.740) and lipid arc classification (Cohen's kappa = 0.823). Interobserver reproducibility for FCT was also good (two-way random-effects, single-measure, absolute-agreement ICC = 0.866).

**Table 2:** OCT characteristics according to TCFA status.

Characteristic	Non-TCFA (*n* = 52)	TCFA (*n* = 63)	*P* value
Fibrous cap thickness, μm	100 (80–130)	60 (40–60)	<0.001
Minimum lumen area, mm^2^	1.69 (1.29–1.97)	1.62 (1.21–2.33)	0.855
Minimum lumen diameter, mm	1.45 (1.27–1.56)	1.43 (1.27–1.71)	0.474
Area stenosis, %	73 (68–77)	76 (66–80)	0.233
Plaque rupture	7 (13.5%)	24 (38.1%)	0.003
Erosion	6 (11.5%)	7 (11.1%)	1.000
Macrophage infiltration	50 (96.2%)	63 (100.0%)	0.202
Microvessels/microchannels	8 (15.4%)	4 (6.3%)	0.135
Calcification	32 (61.5%)	39 (61.9%)	1.000
Layered plaque	5 (9.6%)	11 (17.5%)	0.284
Cholesterol crystals	30 (57.7%)	49 (77.8%)	0.027
Thrombus	8 (15.4%)	21 (33.3%)	0.032

FCT is presented in micrometers to align with the TCFA definition. TCFA was the grouping variable and is not repeated as a table row. Macrophage infiltration denotes an OCT-defined qualitative feature based on signal-rich punctate or band-like regions with signal attenuation rather than quantitative macrophage burden.

OCT, optical coherence tomography; FCT, fibrous cap thickness; MLA, minimum lumen area; MLD, minimum lumen diameter; TCFA, thin-cap fibroatheroma.

Patients with TCFA had thinner fibrous caps than those without TCFA [60 (40−60) vs. 100 (80−130) μm, *P* < 0.001]. The TCFA group also had higher frequencies of plaque rupture (38.1% vs. 13.5%, *P* = 0.003), cholesterol crystals (77.8% vs. 57.7%, *P* = 0.027), and thrombus (33.3% vs. 15.4%, *P* = 0.032). Macrophage infiltration was frequently observed in both groups [63/63 (100.0%) vs. 50/52 (96.2%), *P* = 0.202] and therefore did not discriminate between TCFA and non-TCFA lesions in this ACS and T2DM culprit-lesion cohort. No significant between-group differences were observed in MLA, MLD, area stenosis, erosion, microvessels/microchannels, calcification, or layered plaque.

TCFA was the grouping variable and was not repeated as a table row. Macrophage infiltration denotes an OCT-defined qualitative feature based on signal-rich punctate or band-like regions with signal attenuation rather than quantitative macrophage burden. OCT, optical coherence tomography; FCT, fibrous cap thickness; MLA, minimum lumen area; MLD, minimum lumen diameter; TCFA, thin-cap fibroatheroma.

### Associations between candidate biomarkers and TCFA

The associations between the candidate biomarkers and TCFA are shown in Table 3. In the unadjusted models, TyG, FBG, TG, AIP, and TG/HDL-C were significantly associated with TCFA, whereas HbA1c was not. After adjusting for age, sex, and BMI, these associations remained significant for TyG, FBG, TG, AIP, and TG/HDL-C.

**Table 3:** Associations between core biomarkers and TCFA.

Marker	Model 1 OR (95% CI)	Model 1 P	Model 2 OR (95% CI)	Model 2 P	Model 3 OR (95% CI)	Model 3 P
TyG	3.59 (2.11–6.11)	<0.001	4.02 (2.29–7.06)	<0.001	4.42 (2.34–8.35)	<0.001
FBG	3.18 (1.87–5.42)	<0.001	3.22 (1.87–5.53)	<0.001	3.45 (1.93–6.19)	<0.001
TG	2.54 (1.55–4.16)	<0.001	2.89 (1.70–4.92)	<0.001	2.94 (1.62–5.35)	<0.001
AIP	2.07 (1.34–3.20)	<0.001	2.20 (1.39–3.49)	<0.001	2.08 (1.28–3.40)	0.003
TG/HDL-C	2.04 (1.28–3.23)	0.002	2.14 (1.32–3.46)	0.002	2.04 (1.23–3.39)	0.006
HbA1c	1.35 (0.91–1.99)	0.131	1.34 (0.90–1.99)	0.146	1.35 (0.88–2.08)	0.166

Odds ratios are reported per 1-SD increase in each biomarker. Model 1 was unadjusted. Model 2 adjusted for age, sex, and BMI. Model 3 further adjusted for current smoking, LDL-C, and hypertension. Each biomarker was modeled separately to avoid collinearity among lipid/glucose-derived indices.

In the fully adjusted model, TyG index showed the most consistent association with TCFA (odds ratio per 1-SD increase, 4.42; 95% confidence interval, 2.34−8.35; *P* < 0.001). FBG (odds ratio, 3.45; 95% confidence interval, 1.93−6.19; *P* < 0.001), TG (odds ratio, 2.94; 95% confidence interval, 1.62−5.35; *P* < 0.001), AIP (odds ratio, 2.08; 95% confidence interval, 1.28−3.40; *P* = 0.003), and TG/HDL-C (odds ratio, 2.04; 95% confidence interval, 1.23−3.39; *P* = 0.006) remained associated with TCFA after adjustment. HbA1c levels were not significantly associated with TCFA after adjustment.

In sensitivity analyses, the association between TyG index and OCT-defined TCFA remained consistent after additional adjustment for ACS presentation, calculated eGFR, or log-transformed hs-CRP in separate models (Supplementary Table S2).

Odds ratios were reported per 1-SD increase in each biomarker. Model 1 was unadjusted. Model 2 was adjusted for age, sex, and BMI. Model 3 was further adjusted for current smoking, LDL-C, and hypertension. Each biomarker was standardized and entered into a separate regression model with the same covariate set to avoid collinearity among the lipid/glucose-derived indices. AIP, atherogenic index of plasma; BMI, body mass index; CI, confidence interval; FBG, fasting blood glucose; HDL-C, high-density lipoprotein cholesterol; LDL-C, low-density lipoprotein cholesterol; OR, odds ratio; TCFA, thin-cap fibroatheroma; TG, triglycerides; TyG, triglyceride-glucose index.

### Associations between candidate biomarkers and fibrous cap thickness

The associations between the candidate biomarkers and FCT are summarized in Table 4. FCT was analyzed as a complementary continuous OCT parameter underlying TCFA classification. TyG index showed the largest absolute Spearman rho for its inverse correlation with FCT (rho=−0.530, *P* < 0.001). TG (rho=−0.484, *P* < 0.001), AIP (rho=−0.428, *P* < 0.001), TG/HDL-C (rho=−0.428, *P* < 0.001), and FBG (rho=−0.341, *P* < 0.001) were also inversely correlated with FCT, whereas HbA1c showed only a weak, non-significant correlation.

**Table 4:** Associations between core biomarkers and fibrous cap thickness.

Marker	Spearman rho	Spearman P	Model 3 beta (95% CI), μm	Model 3 P
TyG	−0.530	<0.001	−19.0 (−25.5 to −12.4)	<0.001
FBG	−0.341	<0.001	−10.2 (−16.6 to −3.7)	0.003
TG	−0.484	<0.001	−15.2 (−22.7 to −7.8)	<0.001
AIP	−0.428	<0.001	−12.9 (−19.6 to −6.1)	<0.001
TG/HDL-C	−0.428	<0.001	−9.9 (−17.0 to −2.9)	0.007
HbA1c	−0.171	0.067	−3.7 (−10.4 to 3.0)	0.284

Beta coefficients represent the change in FCT (μm) per 1-SD increase in each biomarker. Model 3 adjusted for age, sex, BMI, current smoking, LDL-C, and hypertension.

In multivariable linear regression, TyG remained inversely associated with FCT after adjustment (*β* per 1-SD increase, −19.0 μm; 95% confidence interval, −25.5 to −12.4 μm; *P* < 0.001). TG, AIP, TG/HDL-C, and FBG remained inversely associated with FCT, whereas HbA1c did not show a stable association.

Beta coefficients represent the change in FCT (μm) per 1-SD increase in each biomarker. Model 3 was adjusted for age, sex, BMI, current smoking, LDL-C, and hypertension. Each biomarker was entered into a separate regression model with the same set of covariates. AIP, atherogenic index of plasma; BMI, body mass index; CI, confidence interval; FBG, fasting blood glucose; FCT, fibrous cap thickness; HDL-C, high-density lipoprotein cholesterol; LDL-C, low-density lipoprotein cholesterol; TG, triglycerides; TyG, triglyceride-glucose index.

### Discriminative ability of candidate biomarkers for TCFA

**Figure 2:**
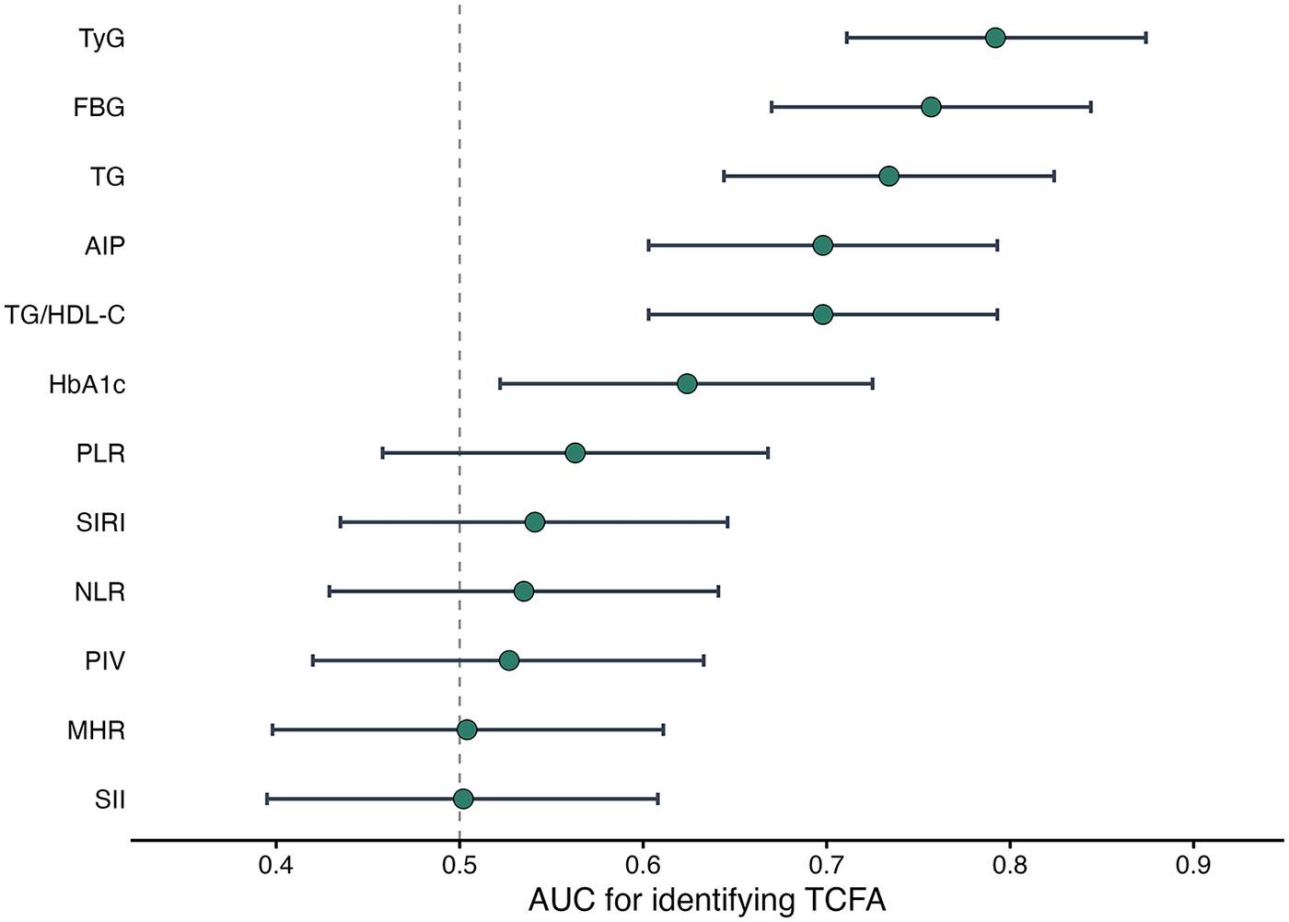
Exploratory single-biomarker discrimination of candidate biomarkers for identifying TCFA. AUC forest plot comparing metabolic and inflammatory indices for identifying OCT-defined TCFA. TyG index showed the largest numerical AUC among the evaluated biomarkers. The AUCs were derived from single-biomarker ROC analyses and should not be interpreted as validation of a clinical prediction model. AIP, atherogenic index of plasma; AUC, area under the curve; FBG, fasting blood glucose; HDL-C, high-density lipoprotein cholesterol; MHR, monocyte-to-HDL-C ratio; NLR, neutrophil-to-lymphocyte ratio; OCT, optical coherence tomography; PIV, pan-immune-inflammation value; PLR, platelet-to-lymphocyte ratio; ROC, receiver operating characteristic; SII, systemic immune-inflammation index; SIRI, systemic inflammation response index; TCFA, thin-cap fibroatheroma; TG, triglycerides; TyG, triglyceride-glucose index.

The discriminative ability of the candidate biomarkers for identifying TCFA is shown in Figure 2. In exploratory single-biomarker ROC analyses, TyG had the largest numerical AUC among all evaluated biomarkers (apparent AUC, 0.792; 95% confidence interval, 0.711−0.874). FBG (AUC, 0.757; 95% confidence interval, 0.670−0.844) and TG (AUC, 0.734; 95% confidence interval, 0.644−0.824) also showed moderate discriminative ability. AIP and TG/HDL-C had identical AUCs of 0.698 (95% confidence interval, 0.603−0.793), while HbA1c showed weaker discriminative ability (AUC, 0.624; 95% confidence interval, 0.522−0.725). Bootstrap optimism correction produced similar estimates and did not materially change the numerical ranking of the core biomarkers (Supplementary Table S1).

Inflammatory indices, including NLR, SII, SIRI, PLR, PIV, and MHR, showed limited discriminative ability for TCFA, with AUCs close to 0.50−0.56. These findings suggest that metabolic indices, particularly TyG, showed numerically higher discrimination than inflammatory indices for identifying OCT-defined TCFA in this cohort.

## Discussion

### Principal findings

In this single-center retrospective study of patients with ACS and T2DM who underwent OCT assessment of one target coronary lesion, several metabolic indices were associated with OCT-defined plaque vulnerability. The main findings were as follows: First, patients with TCFA had higher levels of TG, FBG, HbA1c, TyG, AIP, and TG/HDL-C than those without TCFA, whereas the representative inflammatory indices did not differ substantially between the two groups. Second, TyG index showed the most consistent association with TCFA after multivariable adjustment. Third, TyG was inversely associated with FCT, which is a continuous OCT measure underlying TCFA classification. Finally, TyG had the largest numerical AUC for identifying TCFA among the evaluated metabolic and inflammatory biomarkers in exploratory single-biomarker discrimination analyses.

These findings suggest that in patients with ACS and T2DM, combined glucose-lipid metabolic stress may be more closely linked to OCT-defined TCFA and fibrous cap thinning than systemic inflammatory indices derived from routine blood cell counts. Because FCT is part of the TCFA definition, FCT analysis should be interpreted as a complementary continuous assessment rather than an independent endpoint. The results support further evaluation of TyG as an accessible metabolic correlate of OCT-defined plaque vulnerability, while emphasizing that TyG should not be considered a substitute for intracoronary imaging in clinical practice.

### Comparison with previous studies

The association between TyG and cardiovascular risk has been widely reported, and TyG has been used as a surrogate marker for insulin resistance (8, 9). Prior OCT studies in ACS populations have suggested that a higher TyG index is associated with vulnerable non-culprit plaque characteristics (15). A recent OCT study in patients with stable angina also reported that a higher TyG index was associated with OCT-defined TCFA and high-risk plaque morphology, including thinner fibrous caps and larger lipid arcs (18). Building on this complementary evidence in stable coronary disease, the present study extends this OCT-based evidence line to clinically identified ACS culprit lesions in patients with T2DM, while retaining the need for cautious interpretation because of differences in clinical presentation, lesion context, and TCFA operational definitions.

The present study differs from these prior studies in two important respects. First, it focused specifically on patients with ACS and T2DM, a population in which insulin resistance, dysglycemia, and atherogenic dyslipidemia frequently coexist (5–7). Second, instead of evaluating TyG alone, it compared TyG with several other metabolic and inflammatory indices in the same cohort.

AIP has also been linked to plaque vulnerability in recent OCT studies (16). In the present cohort, AIP was associated with TCFA and FCT; however, its effect estimates and AUC were smaller than those of TyG. Similarly, TG/HDL-C and TG showed meaningful signals, whereas HbA1c was not associated with TCFA in the adjusted models. These findings suggest that integrated glucose-lipid indices may be more closely related to OCT-defined plaque vulnerability than chronic glycemic exposure alone in this cohort.

Selected inflammatory indices, particularly the PIV/SII and PLR, have been investigated in relation to vulnerable plaques and OCT-derived features in previous studies (13, 14). However, in this cohort, the broader exploratory inflammatory panel, including NLR, SII, SIRI, PLR, PIV, and MHR, showed a limited ability to identify TCFA. This does not exclude the role of inflammation in plaque biology, but suggests that routine systemic inflammatory indices may be less informative than metabolic indices for TCFA identification in patients with ACS and T2DM.

The near-universal OCT-defined macrophage signal observed in this cohort should be interpreted as a limitation of this OCT feature for discriminating TCFA status rather than as a group-specific finding. Several factors may account for this high prevalence. First, macrophage infiltration was assessed as a binary qualitative OCT feature based on signal-rich punctate or band-like regions with signal attenuation according to established OCT criteria (3, 4); this definition is sensitive for detecting macrophage-related signal but does not grade severity or quantify macrophage burden. Second, all lesions analyzed in this study were ACS culprit lesions, a setting in which plaque inflammation is expected to be common. Third, the cohort consisted entirely of patients with T2DM, in whom vascular inflammation may be amplified. We therefore cannot exclude the possibility that the high prevalence partly reflects the sensitivity of the binary OCT definition in this high-risk culprit-lesion population rather than a true difference in macrophage biology between the TCFA and non-TCFA groups. Accordingly, macrophage signal was common in both groups and was not useful for discriminating TCFA status in this cohort.

### Potential mechanisms

Several mechanisms may explain the association between TyG index and OCT-derived plaque vulnerability. The TyG index integrates fasting glucose and triglyceride levels and reflects insulin resistance-related metabolic stress (8, 9). Insulin resistance and related metabolic stress have been linked to impaired vascular insulin signaling, endothelial dysfunction, oxidative stress, vascular inflammation, smooth muscle cell dysfunction, and dysregulated lipid handling (19). Hypertriglyceridemia and glucose dysregulation may contribute to lipid accumulation, necrotic core expansion, fibrous cap thinning, and plaque destabilization (5–7, 19).

In patients with T2DM, the coexistence of hyperglycemia, atherogenic dyslipidemia, and insulin resistance may contribute to the formation of vulnerable plaques (5–7). Therefore, TyG may capture a biologically relevant metabolic phenotype that is not fully reflected by HbA1c, TG, or FBG levels alone. The observed inverse association between TyG and FCT is consistent with the link between metabolic disturbance and fibrous cap thinning.

### Clinical and research implications

The TyG index is inexpensive, easily calculated, and based on routine fasting laboratory tests. In patients with ACS and T2DM, the present findings suggest that a higher TyG index is associated with OCT-defined vulnerable plaque features. This association may help generate hypotheses for future prospective studies evaluating glucose-lipid metabolic indices in addition to conventional clinical risk factors. Nevertheless, TyG should be viewed as a complementary research marker rather than a replacement for OCT or comprehensive clinical judgment.

The comparative design of this study was methodologically relevant. Many routine biomarkers are available for patients with ACS, but not all provide equivalent information. The present findings suggest that TyG index showed a more consistent association with OCT-defined TCFA than isolated glycemic markers, AIP, TG/HDL-C, and routine inflammatory indices in this ACS and T2DM cohort.

### Limitations

This study had several limitations. First, it was a single-center retrospective study with a modest sample size, which limits causal inference and external generalizability of the results. Second, patients were included only if they underwent OCT imaging, which may have introduced a selection bias. Third, although the primary model and targeted sensitivity analyses adjusted for several clinical, renal-function, and inflammatory factors, residual confounding remains possible. In particular, detailed pre-admission and longitudinal information on statin therapy, antidiabetic medications, insulin use, treatment changes, and diabetes duration was not consistently available in a standardized analyzable form; therefore, these treatment-related factors could not be fully incorporated into the multivariable models. Fourth, laboratory timing in an ACS cohort may be influenced by acute presentation and early in-hospital management; although TyG was calculated from fasting TG and fasting glucose, fasting samples in emergency PCI patients may have been obtained after clinical stabilization rather than before angiography or PCI. Fifth, multiple biomarkers were compared, and inflammatory analyses should be considered exploratory. Sixth, formal pairwise comparisons of the AUCs were not performed, and the ROC results should be interpreted as exploratory single-biomarker discrimination. Bootstrap optimism correction was performed, but external validation was not available. Seventh, the study assessed OCT-defined plaque vulnerability rather than prospective clinical events; therefore, the findings should not be interpreted as validation of a clinical prediction model or as evidence of clinical utility. Finally, OCT-defined macrophage signal is a qualitative imaging feature and should not be interpreted as quantitative macrophage burden.

## Conclusions

In patients with ACS and T2DM, TyG index showed the most consistent association with OCT-defined TCFA and the continuous FCT component underlying TCFA classification among routinely available metabolic and inflammatory indices. The TyG index also had the largest numerical discriminative estimate for identifying TCFA in exploratory single-biomarker analyses in this cohort. These findings suggest that TyG is an accessible metabolic correlate of coronary plaque vulnerability in this high-risk population, pending confirmation in larger prospective studies.

## Data Availability

The raw data supporting the conclusions of this article will be made available by the authors, without undue reservation.
