# Supplementary material for: Comparative value of metabolic and inflammatory indices for identifying OCT-derived coronary plaque vulnerability in patients with acute coronary syndrome and type 2 diabetes mellitus
Source: Front Cardiovasc Med. 2026 Jul 29;13:1892356. doi: 10.3389/fcvm.2026.1892356 (PMC13461444; doi:10.3389/fcvm.2026.1892356)
Supplement: Supplementary file 1 [file Datasheet1.docx]

Supplementary Material

Manuscript ID: 1892356

Title: Comparative Value of Metabolic and Inflammatory Indices for Identifying OCT-Derived Coronary Plaque Vulnerability in Patients With Acute Coronary Syndrome and Type 2 Diabetes Mellitus

# Supplementary Table S1. Bootstrap optimism-corrected AUCs for the prespecified core biomarkers in exploratory single-biomarker ROC analyses

| **Biomarker** | **Apparent AUC** | **Mean optimism** | **Optimism-corrected AUC** |
| --- | --- | --- | --- |
| TyG | 0.792 | -0.001 | 0.793 |
| FBG | 0.757 | -0.000 | 0.757 |
| TG | 0.734 | 0.003 | 0.732 |
| AIP | 0.698 | -0.002 | 0.700 |
| TG/HDL-C | 0.698 | -0.000 | 0.698 |
| HbA1c | 0.624 | 0.011 | 0.613 |

Note: AUCs were derived from univariable single-biomarker logistic models for OCT-defined TCFA. Bootstrap optimism correction was performed using 1,000 resamples. These analyses were exploratory and were not intended to validate a clinical prediction model.

# Supplementary Table S2. Sensitivity analyses for the association between TyG index and OCT-defined TCFA

| **Model** | **Additional covariate** | **OR per 1-SD increase in TyG index** | **95% CI** | **P value** |
| --- | --- | --- | --- | --- |
| Primary model | None | 4.418 | 2.338-8.349 | <0.001 |
| Sensitivity model 1 | ACS subtype | 4.052 | 2.127-7.720 | <0.001 |
| Sensitivity model 2 | calculated eGFR | 4.415 | 2.339-8.332 | <0.001 |
| Sensitivity model 3 | log-transformed hs-CRP | 4.262 | 2.254-8.056 | <0.001 |

Note: The primary model included TyG index, age, sex, BMI, current smoking, LDL-C, and hypertension. Sensitivity models added the listed covariate one at a time to avoid overparameterization. ACS subtype was modeled using indicator variables with unstable angina as the reference category. eGFR was calculated from age, sex, and serum creatinine using the CKD-EPI 2021 creatinine equation. hs-CRP was log-transformed because of its skewed distribution.
